# Supplementary material for: Agile perching maneuvers in birds and morphing-wing drones
Source: Nat Commun. 2024 Sep 27;15:8330. doi: 10.1038/s41467-024-52369-4 (PMC11437188; doi:10.1038/s41467-024-52369-4)
Supplement: Supplementary file 1 — Supplementary Information [file 41467_2024_52369_MOESM1_ESM.pdf]

Supplementary Information

Agile perching maneuvers in birds and morphing-wing drone

Valentin Wüest[1], Simon Jeger[1], Mir Feroskhan[2], Enrico Ajanic[1], Fabio Bergonti[3], Dario Floreano[1,*]

[1]Laboratory of Intelligent Systems, École Polytechnique Fédérale de Lausanne, Rte Cantonale, Lausanne, 1015, Vaud, Switzerland.

[2]School of Mechanical and Aerospace Engineering, Nanyang Technological University, 50 Nanyang Ave, Singapore, 639798, Singapore, Singapore.

[3]Artificial and Mechanical Intelligence Laboratory, Istituto Italiano di Tecnologia, via San Quirico 19D, Genova, 16163, Genova, Italy.

Contents

Supplementary Fig. 1: Distance of Harris' hawk to pole on impact 2

Supplementary Fig. 2: Comparison of minimization criteria with Harris' hawks flight trajectories 3

Supplementary Fig. 3: Control system schematic 4

Supplementary Fig. 4: Distribution of key characteristic data 4

Supplementary Fig. 5: Varying initial altitude optimizations 5

Supplementary Fig. 6: Drone launcher hardware components 5

Supplementary Fig. 7: Non-dimensional aerodynamic parameters 6

Supplementary Note 1: Consideration of target distance on impact 6

Supplementary Note 2: Explanation of the three minimization criteria 6

Supplementary Note 3: Integration of drone control system 8

Supplementary Note 4: Statistical analysis of characteristic data points 8

Supplementary Note 5: Effect of varying initial altitude on maneuver 8

Supplementary Note 6: Servo dynamics model 9

Supplementary Note 7: Actuator delay compensation 9

Supplementary Note 8: Mechanical design of the drone launcher 9

Supplementary Note 9: Wing sweep for stability modulation on LisEagle 10

Supplementary Table 1: Shift of Center of Gravity and Neutral Point 10

Supplementary Fig. 1: Distance of Harris' hawk to pole on impact

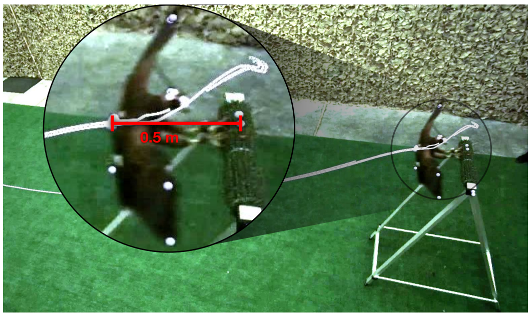

Supplementary Fig. 1: **Distance of Harris' hawk to pole on impact.** Video footage from the Harris' hawk study [3] showing the bird during an agile perching maneuver with overlayed motion-tracking data (zoom image added). The bird's posture and extended legs lead to an approximate distance of 0.5 m to the target position at the moment of impact.

Supplementary Fig. 2: Comparison of minimization criteria with Harris' hawks flight trajectories

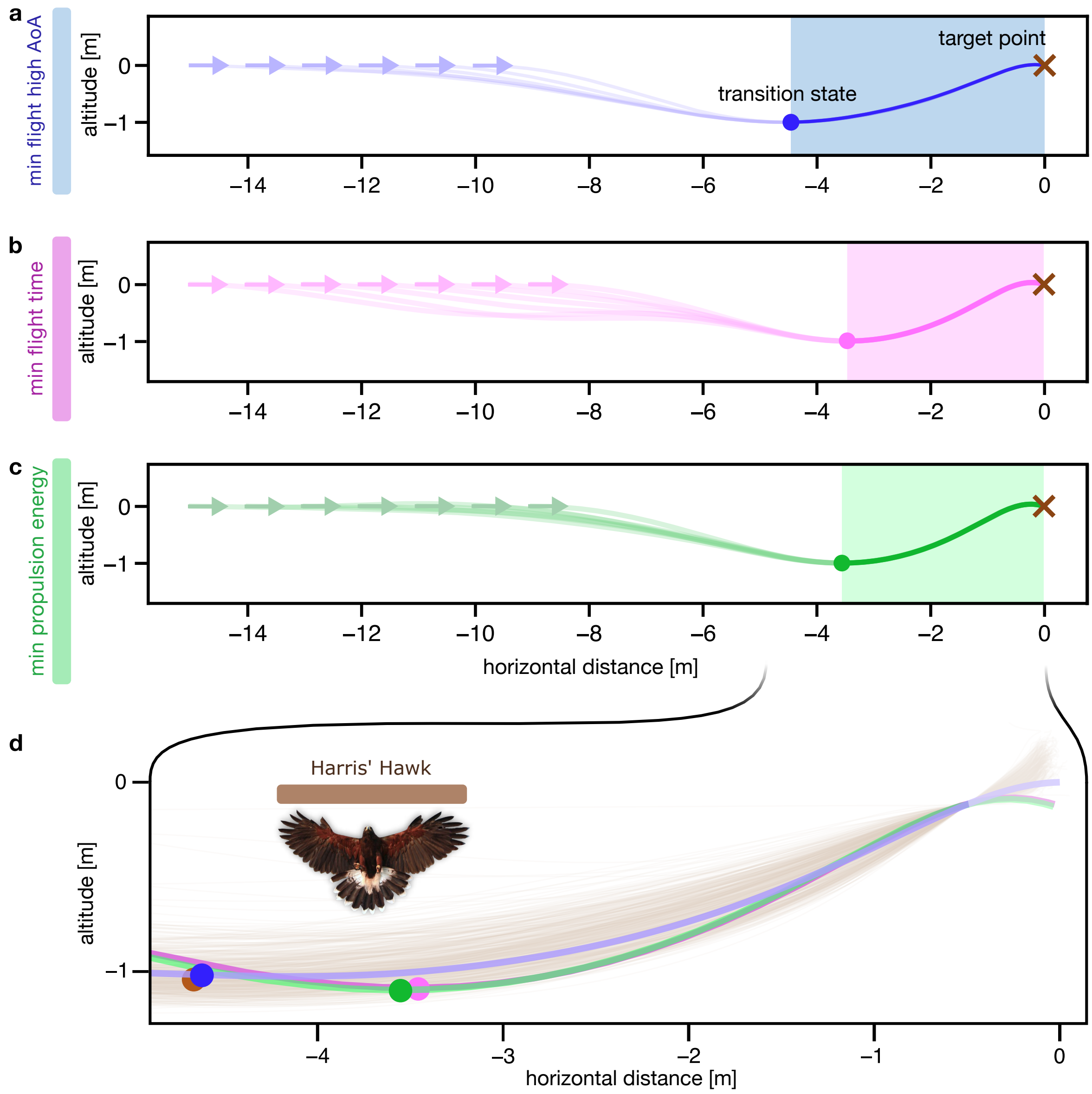

Supplementary Fig. 2: **Comparison of trajectories resulting from three minimization criteria with trajectories displayed by Harris' hawks.** (**a-c**) Perching flight trajectories resulting from three different optimization objectives, which are minimized alongside the final kinetic energy, as described in Methods. Panel (**a**) shows trajectories resulting from minimization of flight distance at high angle of attack (AoA) [3]. We contrast this with trajectories resulting from minimization objectives used in technical applications [5, 6, 10]: (**b**) total flight time and (**c**) propulsion energy. (**d**) Comparison of these three optimization objectives with Harris' hawk flights (depicted in brown), mapping out the transition states (denoted by circles). While flight path and position of the transition state of minimized distance at high angle of attack closely match the recorded Harris' hawk flights (4.1 and 4.2 m from impact respectively), trajectories of the other two criteria transition closer to the impact (3.0 m and 3.1 m from impact respectively) and display a distinctly different flight path. Source data are provided as a Source Data file.

Supplementary Fig. 3: Control system schematic

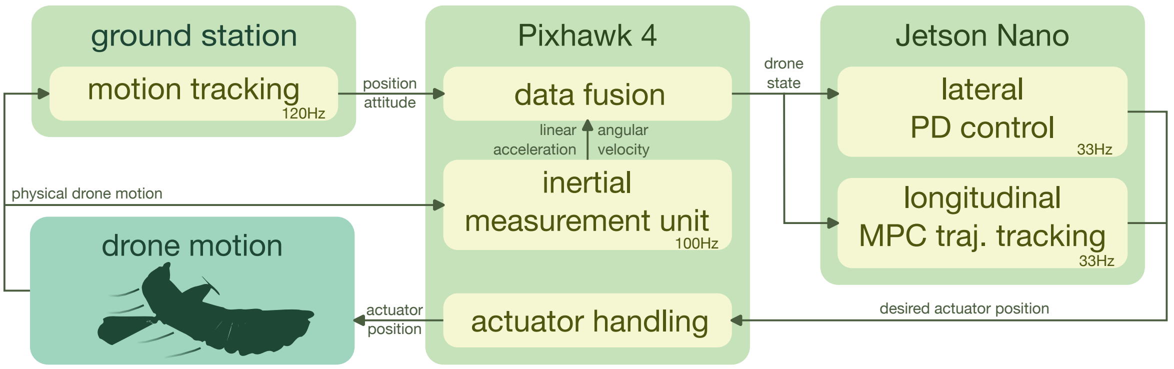

Supplementary Fig. 3: **Control system overview.** The ground-station computer sends measurements to the onboard Pixhawk 4 autopilot, which fuses the information with inertial measurements to estimate the drone state. The estimated state is passed on to the onboard Nvidia Jetson Nano to control lateral motion with Porportional-Derivative (PD) control and longitudinal motion with trajectory tracking control based on Model Predictive Control (MPC). Finally, both controllers on the Nvidia Jetson Nano send the desired actuator positions to the autopilot.

Supplementary Fig. 4: Distribution of key characteristic data

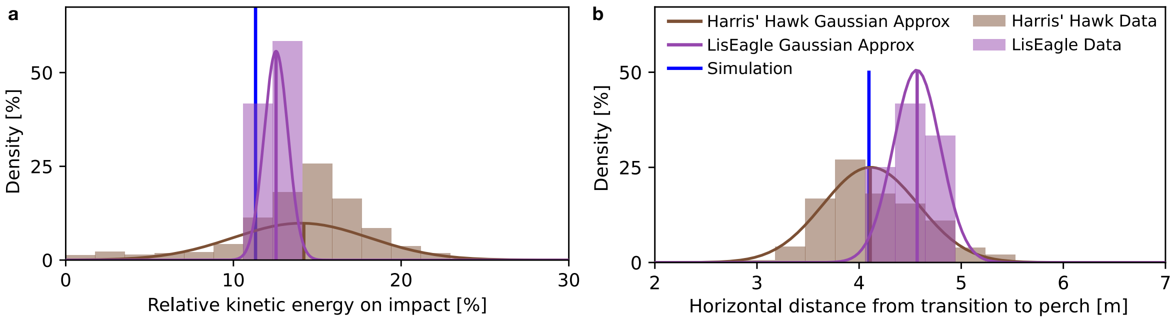

Supplementary Fig. 4: **Distribution of key characteristic data of bird, drone, and simulation flights.** The figures show the distribution of (**a**) the relative kinetic energy on impact and (**b**) the horizontal distance from the transition point to the perch point. We show the histogram of all flights and show an approximation of a Gaussian distribution to help visualize the z-score (see Supplementary Note 7). Source data are provided as a Source Data file.

Supplementary Fig. 5: Varying initial altitude optimizations

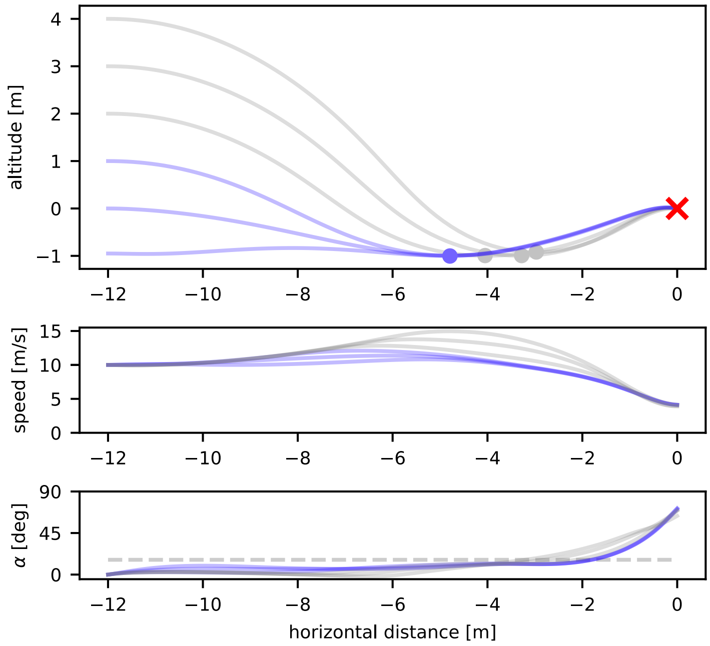

Supplementary Fig. 5: **Perching optimization with varying initial altitude.** Flight trajectories for perching, starting from an initial horizontal distance of 12 m from the target, with initial relative altitude of -0.95, 0, 1, 2, 3, and 4 m. The target is indicated by a red cross and the ground is located at -1 m. We provide a side view of the flight paths and the speed and angle of attack (α) profile over the horizontal position to the target. Circles denote the transition state form the dive to the climb phase at the lowest point of the trajectory, which remains consistent for trajectories starting between -0.95 m and 1 m (shown in blue). As the initial altitude increases beyond 1 m, the transition point shifts upward and closer to the target (shown in grey), increasing the distance allocated to the diving phase. The grey trajectories also exhibit a longer distance flown at higher angles of attack. Source data are provided as a Source Data file.

Supplementary Fig. 6: Drone launcher hardware components

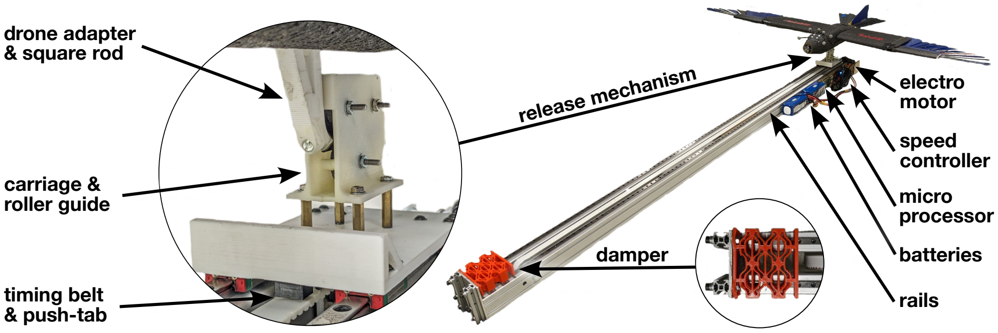

Supplementary Fig. 6: **Drone launcher hardware components.** Release mechanism, consisting of an adapter on the drone's underbelly, where a protruding square carbon rod slots into three concave rollers on a carriage. This mechanism prevents any rotation of the drone, while it is propelled forward by a push-tab on a timing belt. The timing belt is accelerated by a battery-powered electro motor that is controlled by a speed controller, receiving commands from a micro processor. The belt pushes the carriage for 1.8 m along a rail, where the timing belt lowers down and disengages. The carriage is decelerated by a 3D-printed custom damper, leading to the release of the male adapter and initiating drone flight.

Supplementary Fig. 7: Non-dimensional aerodynamic parameters

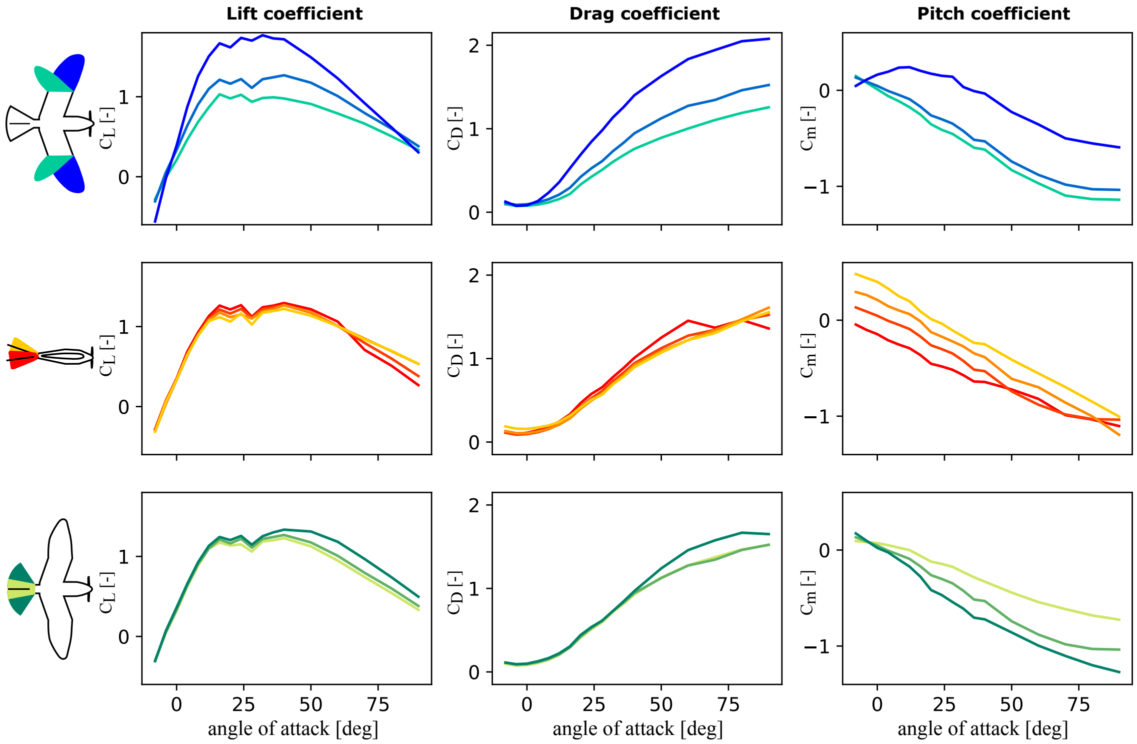

Supplementary Fig. 7: **Representation of the non-dimensional aerodynamic coefficients affected by changing actuator positions.** Representation of values within the coefficient matrix showing lift coefficients in the left column, drag coefficients in the middle column, and pitch coefficients in the right column. Each row represents the changes of the coefficients along a degree of freedom, where the wing sweep changes are on the top row, tail incidence changes in the middle row, and tail sweep in the bottom row. In each cell, the plot colors represent the actuator positions at min, central, and max position, as described in Methods. Source data are provided as a Source Data file.

Supplementary Note 1: Consideration of target distance on impact

In the perching maneuvers of Harris' hawks, the impact occurs before their center of gravity reaches the pole at the target point. As they touch the pole, energy is dissipated through their legs. For our analysis, it is crucial to assess the energy redistribution exclusively during the flight phase, before the impact. As a result, we evaluate their flights right before the point of impact. In Supplementary Fig. 1, we show how we deduce this distance based on video footage overlayed with motion tracking data from Supplementary Material of the Harris' hawk study [3]. The posture and leg extension of the birds suggest an approximate distance of 0.5 m identified by finding the corresponding point in the provided motion tracking data. Consequently, it is at this point that we evaluate the distance required for the climb phase and energy redistribution analysis. We take this into account also in our simulations and drone experiments by evaluating these metrics 0.5 m before the target point is reached.

Supplementary Note 2: Explanation of the three minimization criteria

It has been hypothesized that the perching control strategy displayed by birds aims at minimizing not only impact energy but also distance flown at high angle of attack [3]. In contrast, previous robotic implementations of the perching maneuver with winged

drones minimized either flight time or energy used for propulsion [5, 6, 10]. In addition to the experiments with minimization of distance flown at high angles of attack described in the main text, we also conducted two sets of control experiments that minimize total flight time and energy used for propulsion, respectively. For each experiment, we apply the same optimization procedure and experimental settings described in the Methods section of the manuscript, including the minimization of the final kinetic energy, as described in Equation 7 of the main text.

Minimization of flight time at high angle of attack

To recreate the hypothesized avian strategy, we aim to minimize the distance flown at high angle of attack. As described in the Methods section, we define the corresponding smooth cost at step k as

$$\tilde{\mathbf{L}}_{\alpha,k} = V_k \Delta t \cdot \left(\tfrac{1}{2} \tanh \left(\frac{\alpha_k - \alpha_{\text{stall}}}{\epsilon} \right) + \tfrac{1}{2} \right) \tag{1}$$

where α_{stall} is the defined stall angle of $\alpha_{\text{stall}} = 16°$.

Minimization of total flight time

In order to minimize time to impact, we replace $\tilde{\mathbf{L}}_{\alpha,k}$ with a cost on the optimization variable Δt, such that the cost at step k is defined as

$$\mathbf{L}_{\Delta t,k} = \Delta t \tag{2}$$

Minimization of propulsion energy

To minimize propulsion energy, we require a power consumption model. We thus fix the motor with propeller on a thrust stand, measure thrust and corresponding power consumption across the motor's entire operation range, and fit a quadratic function $p\left(a_{\text{th},k}\right)$ to it providing us with a power consumption estimate based on the currently applied thrust. The consumed propulsion energy cost at time k can thus be defined as:

$$\mathbf{L}_{\text{prop},k} = \Delta t \cdot p\left(a_{\text{th},k}\right), \quad \text{with} \quad p\left(a_{\text{th},k}\right) = c_0 + c_1 \cdot a_{\text{th},k} + c_2 \cdot a_{\text{th},k}^2 \tag{3}$$

Resulting trajectories

Compared to the trajectories resulting from minimization of flight at high angle of attack (Supplementary Fig. 2a), the trajectories resulting from the minimization of total flight time (Supplementary Fig. 2b) display a different flight path where the transition to the climb phase occurs closer to the perching point (3.0 m vs 4.1 m). Similarly, the trajectories resulting from the minimization of the propulsion energy (Supplementary Fig. 2c) transition to the climb phase closer to the perching point (3.1 m. vs 4.1 m).

A comparison of the trajectories resulting from those three minimization criteria with the trajectories of Harris' hawks (Supplementary Fig. 2d) shows that the minimization of flight at high angles of attack produces flight paths with transition points very similar to those of the birds (4.1 m vs 4.2 m respectively). These control experiments further corroborate the hypothesis that birds may prioritize control authority over flight time or invested propulsion energy during agile maneuvers.

Supplementary Note 3: Integration of drone control system

In the drone control system, there are multiple processors working together. On the drone, there is a Nvidia Jetson Nano [7] with a custom carrier board [4] that orchestrates the control and a Pixhawk 4 autopilot [9] that runs the estimator and controls the actuator. An overview of the involved components and the flow of information are shown in Supplementary Fig. 3. On the ground, there is a laptop that sends motion-tracking data over WiFi through the Robotic Operating System (ROS) [8] to the drone. On the drone, the autopilot fuses this information with measurements from its onboard Inertial Measurement Unit (IMU) to obtain estimations of its position, attitude, linear, and angular velocity. These estimates are sent to the Jetson Nano, where the trajectory tracking control and reactive controllers (see Methods) calculate the commanded actuator positions. These commands are subsequently sent back to the autopilot which controls the servo positions.

Supplementary Note 4: Statistical analysis of characteristic data points

In our experiments we obtain two characteristic metrics of a perching maneuver, the relative kinetic energy on impact and horizontal distance from the transition point to the perch. To compare how closely the simulation and drone experiments match the observed Harris' hawk flight behavior, we provide their distribution in Supplementary Fig. 4.

Firstly, we analyze how closely the simulation and drone experiments match the bird behavior. As Supplementary Fig. 4 demonstrates, we find that both simulation and drone experiments lie within the distribution of the Harris' hawk flights and are statistically close. To quantify this statement, we further calculate the z-score (a metric reporting the deviation of a measurement from the mean, measured in standard deviations) of the simulation and mean of the LisEagle flights within the distribution of Harris' hawk flights. We find a z-score of -0.56 and -0.03 for the simulation and -0.33 and 0.75 for the LisEagle flights for the kinetic energy and horizontal distance, respectively. The z-scores thus indicate that all metrics lie within less than a standard deviation from the mean of Harris' hawk flights. This close correlation is an indication that the drone experiments capture the key characteristics of the bird flight.
Secondly, we observe that the standard deviation of the drone experiments is smaller than that of the bird experiments. We find standard deviations of 0.72 % and 0.23 m on the drone, compared to 4.06 % and 0.47 m on the Harris' hawk for the relative impact energy and required horizontal distance. As we mention in the manuscript, the decreased standard deviation allows hypothesis validation with fewer samples, reducing the need for a large set of experiments.

Supplementary Note 5: Effect of varying initial altitude on maneuver

Although in this article we aimed at replicating the experimental conditions used in bird studies [1, 3], we also used the method to investigate perching maneuvers starting from different altitudes compared to the perching point, a situation that birds may encounter in nature. Specifically, we used the same conditions described in the manuscript, *i.e.* the drone started in straight flight condition 12 m away from the target at the nominal speed of 10 ms^{-1}, the target was located at 0 m altitude, and the drone was not allowed to touch the ground at -1 m during the dive phase. Given these constraints and the dynamics of our avian-inspired drone, we minimized impact energy and distance flown at high angle of attack for different initial altitudes, namely

-0.95, 0, 1, 2, 3, and 4 m relative to the target altitude).
The results shown in Supplementary Fig. 5 indicate that the drone could reduce the kinetic energy at impact for all altitude variations. However, starting altitudes higher than 1 m relative to the target point resulted in longer horizontal distance allocated to the dive phase and in longer distance flown at high angles of attack in the climb phase. Whether these maneuvers are adopted also by birds at those altitudes remains to be studied.

Supplementary Note 6: Servo dynamics model

To capture the limitations of the actuators, we model each servo as a Proportional-Derivative (PD) controlled second-order system with limited acceleration and limited velocity. Here, we describe the dynamics of one of the servos with its state $\mathbf{x}_{\mathrm{a}} = [a, \dot{a}]$, consisting of its normalized angular position a and angular velocity $\dot{a}$. The control input of the system $\mathbf{u}_{\mathrm{a}} = \bar{a}$ is its desired angular position, which results in the dynamics as

Algorithm 1 Calculate servo dynamics

calculate applied acceleration:
$\ddot{a} \leftarrow K_{\mathrm{p}}(\bar{a} - a) + K_{\mathrm{d}}(\text{-}\,\dot{a})$
$\ddot{a} \leftarrow \mathrm{clamp}(\ddot{a}, \text{-}\,\ddot{a}_{\max}, \ddot{a}_{\max})$

simulate for Δt :
$a \leftarrow a + \dot{a}\,\Delta t$
$a \leftarrow \mathrm{clamp}(a, 0, 1)$
$\dot{a} \leftarrow \dot{a} + \ddot{a}\,\Delta t$
$\dot{a} \leftarrow \mathrm{clamp}(\dot{a}, \text{-}\,\dot{a}_{\max}, \dot{a}_{\max})$

As mentioned in Methods, we obtain all parameters K_p, K_d, $\ddot{a}_{\max}$, and $\dot{a}_{\max}$ from data we recorded in the motion capture system by sending steps between 0 and 1 at different frequencies (more details in Supplementary Material Code [11]).

Supplementary Note 7: Actuator delay compensation

We apply Smith Compensation (SC) to account for the compound delay as explained in Methods. By applying SC, we obtain the robot body and actuator states at the point, where the currently sent control inputs start affecting the dynamics, i.e. $\Delta t_{\mathrm{delay}} = 0.09\,\mathrm{s}$ in the future.

To achieve this, we keep a history of actuator commands sent in the past for the duration of the delay. Concurrently, we simulate the actuator dynamics to obtain an estimate of their current angular position and velocity, providing us with an estimate of the current system state $\mathbf{x}_{\mathrm{mpc}}$. The system state and actuator command history allow us to simulate the system for the duration of $\Delta t_{\mathrm{delay}}$ to obtain the state, where the control inputs start affecting the system $\mathbf{x}_{\mathrm{mpc,pred}}$. Finally, we initialize the NMPC optimization process with $\mathbf{x}_{\mathrm{mpc,pred}}$.

Supplementary Note 8: Mechanical design of the drone launcher

To ensure repeatable deployment into the transition state, the drone is deployed by a custom-built launcher (design inspired from [12]). A micro processor (Raspberry Pi 4) sends commands to a speed controller (ODrive Pro) that regulates the speed of an electro motor (ODrive D6374 150 KV, 2.3 kW) through the power provided by two batteries (4000 mAh, 6 S, 60 C Lithium Polymer (LiPo) batteries) to propel a

4 m long driving belt. A push-tab on the driving belt links to a carriage, which is guided by four linear bearings along a 2 m long double rail (Supplementary Fig. 6). Positioned centrally above the double rail is a carriage that features a linear guide consisting of three concave rollers. The rollers accommodate a horizontally extended square carbon fiber rod that protrudes from an adapter at the drone's underbelly, securing the drone and preventing any rotational movement during the acceleration. Over a 1.8 m distance, the launcher propels the drone to a predetermined velocity, here $11.6 \pm 0.3\,\mathrm{ms^{-1}}$, where the push-tab disengages the carriage. At the end of the rail, the carriage is decelerated by a custom 3D-printed Thermoplastic Polyurethane (TPU) damper. Concurrently, the drone disengages from the decelerating adapter and flies off into the transition state of the perching trajectory, which corresponds to the beginning of the agile climb phase.

Supplementary Note 9: Wing sweep for stability modulation on LisEagle

The relative position of Center of Gravity (CoG) and Neutral Point (NP) is a significant characteristic of an aircraft's stability. Birds have been shown to take advantage of this relation by sweeping their wings to modulate between stable and unstable flight configurations. Gulls for example are believed to increase their stability in gusty conditions and reduce stability to achieve higher agility [2]. However, by sweeping their wings forward, both the CoG and NP advance. In light of this insight, we studied the shift of CoG and NP on our avian-inspired drone and reported them in Supplementary Tab. 1, where we assume central positions for all other actuators and flight at its designed flight speed of $10\,\mathrm{ms^{-1}}$. We observe that the CoG shifted 2.5 cm (from -1.3 cm to 1.2 cm) and the NP shifted 11.1 cm (from -2.7 cm to 8.4 cm) forward. This leads to a relative shift of 8.6 cm from the NP being behind the CoG (-1.4 cm) to it is far in front of it (7.2 cm). This shift implies that the drone's flight stability characteristics are considerably changed when sweeping from a collapsed to an extended wing configuration.

Supplementary Table 1: Shift of Center of Gravity and Neutral Point

**configuration**	**wing sweep angle** [°]	**CoG position** [cm]	**NP position** [cm]	**relative position** [cm]
**collapsed**	45.0	-1.3	-2.7	-1.4
**central**	87.5	0.0	1.6	1.6
**extended**	130.0	1.2	8.4	7.2

Supplementary Tab. 1: Center of Gravity (CoG) and Neutral Point (NP) position given wing sweep in its collapsed, central, and extended state. We also report their relative position, as it provides crucial insight into the aircraft's stability characteristics. Here, we have set the other actuators at their central position and focused solely wing sweep.

References

[1] Carruthers AC, Thomas AL, Walker SM, et al (2010) Mechanics and aerodynamics of perching manoeuvres in a large bird of prey. The Aeronautical Journal 114(1161):673–680. https://doi.org/10.1017/s0001924000004152

[2] Harvey C, Baliga V, Goates C, et al (2021) Gull-inspired joint-driven wing morphing allows adaptive longitudinal flight control. Journal of the Royal Society Interface 18(179):20210132. https://doi.org/10.1098/rsif.2021.0132

[3] KleinHeerenbrink M, France LA, Brighton CH, et al (2022) Optimization of avian perching manoeuvres. Nature 607(7917):91–96. https://doi.org/10.1038/s41586-022-04861-4

[4] Laboratory of Intelligent Systems - EPFL (2023) LIS Vision Flight Hardware: Jetson Nano Carrier Board. https://github.com/lis-epfl/lis-vision-flight/tree/main/hardware#jetson-nano-carrier-board

[5] Moore J, Cory R, Tedrake R (2014) Robust post-stall perching with a simple fixed-wing glider using lqr-trees. Bioinspiration & biomimetics 9(2):025013

[6] Novati G, Mahadevan L, Koumoutsakos P (2019) Controlled gliding and perching through deep-reinforcement-learning. Physical Review Fluids 4(9):093902

[7] NVIDIA Corporation (2023) Nvidia jetson nano developer kit. URL https://developer.nvidia.com/embedded/jetson-nano-developer-kit, accessed: 2023-12-07

[8] Open Source Robotics Foundation (2023) Ros (robot operating system). URL https://www.ros.org/, accessed: 2023-12-07

[9] Pixhawk (2023) Pixhawk 4. URL https://docs.px4.io/master/en/flight_controller/pixhawk4.html, accessed: 2023-12-07

[10] Waldock A, Greatwood C, Salama F, et al (2018) Learning to perform a perched landing on the ground using deep reinforcement learning. Journal of intelligent & robotic systems 92:685–704

[11] Wüest V, Jeger S, Feroskhan M, et al (2023) Agile perching maneuvers in birds and morphing-wing drones. https://doi.org/10.5281/zenodo.10283445

[12] Zufferey R, Tormo-Barbero J, Feliu-Talegón D, et al (2022) How ornithopters can perch autonomously on a branch. Nature Communications 13(1):7713. https://doi.org/10.1038/s41467
