# Supplementary material for: Agile perching maneuvers in birds and morphing-wing drones
Source: Nat Commun. 2024 Sep 27;15:8330. doi: 10.1038/s41467-024-52369-4 (PMC11437188; doi:10.1038/s41467-024-52369-4)
Supplement: Supplementary file 3 — Description of Additional Supplementary Files [file 41467_2024_52369_MOESM3_ESM.pdf]

**Description of Additional Supplementary Files**

**Supplementary Data 1**
Code and dataset of recorded experiments, allowing to recreate simulation experiments and conduct flight experiment data analysis of drone and bird to arrive at the information shown in the manuscript.

**Supplementary Movie 1 - Flight phases of the perching maneuver**
A video illustrating the flight phases of the perching maneuver discussed in this paper. Flight footage adapted from KleinHeerenbrink et al., Nature, 2022, under a CC-BY 4.0 license.

**Supplementary Movie 2 - Morphing actuation**
Illustration of the drone's degrees of freedom, highlighting those used for longitudinal and lateral control.

**Supplementary Movie 3 - Optimal drone trajectory**
Demonstration of resulting optimal drone trajectory in simulation and the energy redistribution achieved during the agile climb phase.

**Supplementary Movie 4 - Comparison simulation, drone, bird**
Side-by-side comparison of simulation to drone experiments and simulation to bird experiments. Each simulation is captured from the same camera angle as its corresponding real-world footage. Flight footage adapted from KleinHeerenbrink et al., Nature, 2022, under a CC-BY 4.0 license.

**Supplementary Movie 5 - Drone experiments**
Video recording of drone perching maneuver experiments showing the flights of multiple trials in real-time and in slow-motion
